# The G8 screening tool enhances prognostic value to ECOG performance status in elderly cancer patients: A retrospective, single institutional study

**DOI:** 10.1371/journal.pone.0179694

**Published:** 2017-06-22

**Authors:** Masahiro Takahashi, Masanobu Takahashi, Keigo Komine, Hideharu Yamada, Yuki Kasahara, Sonoko Chikamatsu, Akira Okita, Shukuei Ito, Kota Ouchi, Yoshinari Okada, Hiroo Imai, Ken Saijo, Hidekazu Shirota, Shin Takahashi, Takahiro Mori, Hideki Shimodaira, Chikashi Ishioka

**Affiliations:** 1 Department of Medical Oncology, Tohoku University Hospital, Aoba-ku, Sendai, Miyagi, Japan; 2 Department of Clinical Oncology, Institute of Development, Aging and Cancer, Tohoku University, Aoba-ku, Sendai, Miyagi, Japan; Baylor University Medical Center, UNITED STATES

## Abstract

**Background:**

Some elderly cancer patients, even with good Eastern Cooperative Oncology Group performance status (ECOG-PS), have poor survival outcomes and cannot tolerate standard therapy. Few studies have detailed the associations between the G8 screening tool, ECOG-PS, and overall survival (OS) in such patients.

**Methods:**

Cancer patients, aged 70 years or older, were assessed for G8 and classified into three groups according to their G8 score: <11 as the low score group, 11–14 as the intermediate score group, and >14 as the high score group. We retrospectively analyzed the association between G8 score and OS in all patients and for each ECOG-PS-categorized group.

**Results:**

Out of 264 enrolled patients, most patients (87%) with solid tumor were categorized as TNM stage IV. ECOG-PS was 0 or 1 in 215 patients and ≥2 in 48; there was missing data for one patient. Among all patients, the low score group with a median OS of 7.7 months survived significantly less than both the high score group with a median OS of 25.6 months [Hazard ratio (HR) 3.48; 95% confidence interval (CI), 1.96–6.63; p < 0.0001] and the intermediate score group with a median of 15.6 months (HR 1.83; 95% CI, 1.28–2.65; p < 0.001). In the multivariate analysis, TNM stage and G8 score were independent prognostic factors for OS. When patients with an ECOG-PS of 0 or 1 were analyzed, patients with a lower G8 score showed significantly shorter OS than patients with a higher score when any two groups were compared.

**Conclusion:**

This novel classification of the G8 score contributes to prompt identification of patients with poor prognosis and improved the prognostic value of ECOG-PS. Using G8 with ECOG-PS may be helpful in deciding treatment for elderly patients with advanced cancer.

## Introduction

The risk of cancer increases with aging. In Japan, approximately 60% of newly diagnosed cancer cases occur in patients aged 70 years or older [1]. The elderly cancer population is heterogeneous, and chronological age alone does not reflect heterogeneity in the aging process. The International Society of Geriatric Oncology recommends that elderly cancer patients should be evaluated for a geriatric assessment (GA) to detect problems not readily identified by routine physical examinations or medical history, to predict cancer treatment-related toxicities, to predict survival, and to assist in cancer treatment decisions [2]. GA is a multidimensional and interdisciplinary evaluation tool that leads to identification of functional, nutritional, cognitive, psychological, social support, and comorbidity factors [3, 4]. Although GA is valuable in oncology, a full GA is time-consuming. Geriatric screening tools such as G8 [5–7], Vulnerable Elders Survey-13 [7, 8], and the Flemish version of the Triage Risk Screening Tool [6, 9] are recommended to identify patients in need of further evaluation via a full GA [10, 11].

The G8 screening tool consists of seven items dealing with food intake, weight loss, mobility, neuropsychological problem, body mass index, prescription drug, and self-perception of health, from the Mini-Nutritional Assessment (MNA) questionnaire and was developed specifically for elderly cancer patients [5]. G8 takes 3–5 minutes. A total score ranges from 0 (poor score) to 17 (good score), and a score of ≤14 is considered abnormal [5]. Sensitivity typically ranges from 65%–92% and has been shown to be more than 80% in many studies detecting impairments via a full GA [10]. Moreover, several studies reported that G8 has prognostic value regarding survival outcomes in elderly patients with various cancers [6, 12] and hematological malignancies [13]. The cut-off value of G8 was set as 14 points in these studies [6,12,13]. However, it is still unclear whether G8 classification into two groups with a cut-off value of 14, particularly in elderly cancer patients with poor prognosis, is adequate to yield an insight into overall survival (OS).

The Eastern Cooperative Oncology Group performance status (ECOG-PS) has been widely used to assess functional status in cancer patients [14]. Patients with poor ECOG-PS survived less than patients with good ECOG-PS in most clinical trials [15–17]. Therefore, ECOG-PS is considered an important prognostic factor employed to make treatment decisions in daily practice. Elderly patients with good ECOG-PS scores are generally considered fit and able to receive standard intensive therapy comparable to younger patients. However, these elderly patients are also heterogeneous. Some patients, even with good ECOG-PS, have poor survival outcomes and/or cannot tolerate standard therapy. There is little data dissecting the association between G8, ECOG-PS, and OS in elderly cancer patients, and thus more research is needed to best identify prognostic factors in order to propose appropriate treatments for individuals with geriatric problems. The purpose of this study was to clarify whether G8 has independent prognostic value for elderly cancer patients, what the optimal cut-off value for predicting prognosis in elderly cancer patients is, and whether G8 enhances the prognostic value of ECOG-PS in elderly cancer patients.

## Materials and methods

### Patient population

Between February 2014 and March 2016, patients aged 70 years or older with malignant tumor, including carcinoma, sarcoma, hematological malignancy, melanoma, pseudomyxoma, mesothelioma, and neuroendocrine tumor at any stage, who presented at the Department of Medical Oncology, Tohoku University Hospital in Japan, were enrolled in this study. This study was approved by the ethics committee of Tohoku University Hospital (2015-1-175). Because all data were accessed anonymously, the committee waived the need for consent from the participants.

### G8 assessment and data collection

The MNA is available in Japanese and used widely in the Japanese general geriatric population [18]. We used the Japanese version of the MNA without changing it to create the Japanese version of G8. Because polypharmacy is defined as more than four drugs in the Japanese version of the MNA, we also assessed polypharmacy as per the MNA definition in item 6 of G8. The G8 screening tool is shown in S1 Table. All patients who participated in this study were assessed for G8 by physicians. A score of >14 was defined as normal and ≤14 was defined as abnormal according to the conventional classification [5]. Furthermore, to clarify whether subclassification of the G8 score can more efficiently identify patients with poor prognosis, we classified all patients into three groups according to their G8 score: a score of 0–10.5 as the low score group, a score of 11–14 as the intermediate score group, and a score of 14.5–17 as the high score group. The high score group was the same as the normal group in the conventional classification scheme. We retrospectively collected patient characteristics, G8 scores, and survival data. Comorbidity was measured via the Charlson comorbidity index (CCI) [19].

### Statistical analysis

All statistical analyses were performed with JMP^®^ 12 (SAS Institute Inc., Cary, NC, USA). The association between G8 score and patient characteristics was analyzed by the Fisher’s exact test. OS was defined as the time from assessment for G8 to the date of death from any cause or censored at the last follow-up date, and OS was estimated via the Kaplan-Meier method. Comparisons of OS data between two groups were performed by the log-rank test. We used the Cox proportional regression hazard model to examine predictors of OS. Covariates with a p-value < 0.10 in the univariate analysis were included in the multivariate analysis. A value of p < 0.05 was considered as statistically significant in all analyses.

## Results

### Patient characteristics

Between February 2014 and March 2016, a total of 267 patients were enrolled in this study. All patients were evaluated using the G8 screening tool. There was an absence of score in one or more items of the G8 in three patients. The remaining 264 patients were evaluated for further survival analyses. Patient characteristics are listed in Table 1. Median age was 75 years (range: 70–91 years), and 21.6% of patients were older than 80 years. ECOG-PS was 0 in 97 patients (36.7%), 1 in 118 (44.7%), and ≥2 in 48 (18.2%) patients; there was missing data for one patient. CCI was 0 in 186 patients (70.5%), 1 in 61 (23.1%), and 2 or more in 17 (6.4%) patients. Tumor types were carcinoma in 229 (86.7%) patients, sarcoma in 17 (6.4%), hematological malignancy in 5 (1.9%), and other malignant tumors in 13 (4.9%) patients. The most common carcinoma sites were the esophagus (n = 58), stomach (n = 40), colon/rectum (n = 36), and pancreas (n = 34). Most of patients enrolled in this study had advanced stage cancer; particularly, 226 (87.3%) of 259 patients with solid tumor were categorized as TNM stage IV. All clinicopathological data of 264 patients enrolled in this study are shown in S2 Table.

**Table 1:** Patient characteristics.

Characteristic	No. of patients (n = 264)	%
Age, years		
Median	75	
Range	70–91	
Age distribution		
70–74	121	45.8
75–79	86	32.6
80–84	45	17.0
≥85	12	4.5
Sex		
Male	174	65.9
Female	90	34.1
ECOG-PS		
0	97	36.7
1	118	44.7
2	25	9.5
3	18	6.8
4	5	1.9
Unknown	1	0.4
Charlson comorbidity index		
0	186	70.5
1	61	23.1
≥2	17	6.4
Tumor type		
Carcinoma		
Esophageal	58	22.0
Gastric	40	15.2
Colorectal	36	13.6
Pancreatic	34	12.9
Bile tract	17	6.4
Unknown primary	15	5.7
Head and neck	13	4.9
Others ^a^	16	6.1
Sarcoma	17	6.4
Hematological malignancy	5	1.9
Other malignant tumors ^b^	13	4.9
TNM stage of solid tumor, n = 259		
I	0	0
II	7	2.7
III	25	9.7
IV	226	87.3
unknown	1	0.4

ECOG-PS denotes Eastern Cooperative Oncology Group performance status.

^a:^ Other carcinomas include breast, lung, thyroid, duodenal, anal canal, renal cell, urothelial, prostate, and ovarian carcinoma.

^b:^ Other malignant tumors include mesothelioma, malignant melanoma, pseudomyxoma, and neuroendocrine tumor.

### G8 score

Median G8 score was 11 (range: 1.5–17); 83.0% of patients had an abnormal score (≤14) (Table 2). More than 50% of patients had a low score in 5 items (food intake, weight loss, body mass index, prescription drug, and self-perception of health), whereas less than 25% of patients had a low score in mobility, neuropsychological problem, and age. Median prescription drug use assessed in item 6 was 5 (range: 0–15), and 12.8% of patients were prescribed 10 or more drugs. The detailed score in each item is shown in S3 Table.

**Table 2:** G8 scores.

G8 score	Points	
Median	11	
Mean	11.2	
Range	1.5–17	
Total score, no. (%)		
Normal (>14)	45	(17.0)
Abnormal (≤14)	219	(83.0)

The associations between G8 score and patient characteristics are shown in Table 3. No significant differences were observed regarding sex, CCI, and stage of solid tumor between patients with an abnormal G8 score and patients with a normal G8 score. The ratio of an abnormal G8 score was significantly higher in patients aged 80 or older than those aged less than 80 (p < 0.01). All 45 patients with a normal G8 score possessed an ECOG-PS of 0 or 1, and no patient with a normal G8 score had an ECOG-PS of ≥2 (p < 0.001).

**Table 3:** Association between G8 scores and patient characteristics.

Characteristic	Total No. of patients (n = 264)	Normal G8 score (>14) No. of patients (n = 45)	Abnormal G8 score (≤14) No. of patients (n = 219)	p value
Age, n = 264				
<80	207	42	165	
≥80	57	3	54	< 0.01
Sex, n = 264				
Male	174	31	143	
Female	90	14	76	0.73
ECOG-PS, n = 263				
0/1	215	45	170	
2/3/4	48	0	48	< 0.001
CCI, n = 264				
0	186	30	156	
≥1	78	15	63	0.59
TNM stage of solid tumor, n = 258				
II/III	32	5	27	
IV	226	40	186	1.00

ECOG-PS denotes Eastern Cooperative Oncology Group performance status.

CCI denotes Charlson comorbidity index.

### OS analyses

**Fig 1:**
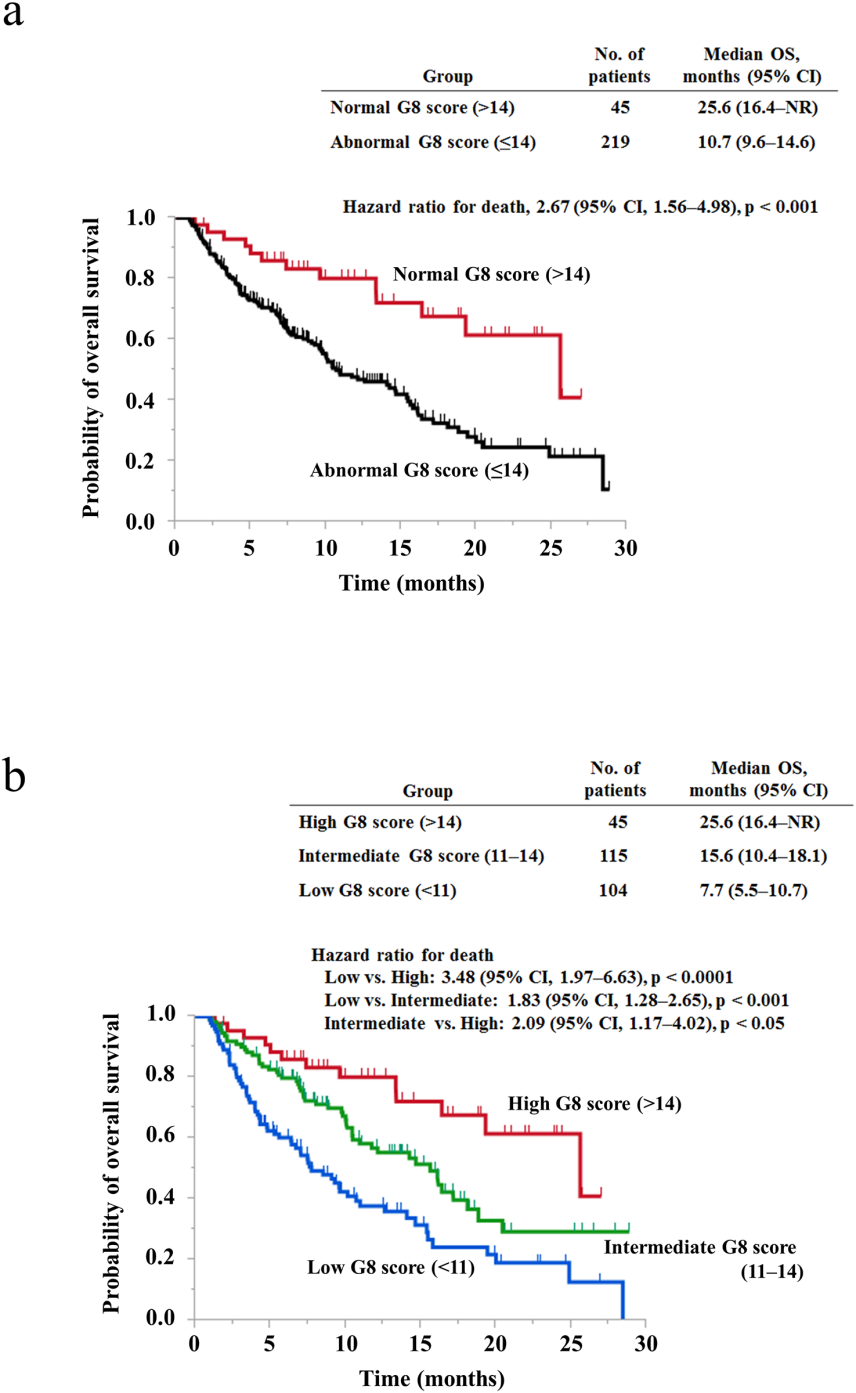
Overall survival according to the G8 score in elderly cancer patients. (a) Kaplan–Meier analyses for overall survival in patients with a normal G8 score (>14) or an abnormal G8 score (≤14). (b) Kaplan–Meier analyses for overall survival in patients with high G8 scores (>14), intermediate G8 scores (11–14), or low G8 scores (<11). NR, not reached.

We compared OS between two groups, patients with an abnormal G8 score and patients with a normal G8 score, as conventionally classified. Median OS was 10.7 months (95% CI, 9.6–14.6) in patients with an abnormal G8 score and 25.6 months (95% CI, 16.4 to not reached) in patients with a normal G8 score [hazard ratio (HR) for death, 2.67; 95% confidence interval (CI), 1.56–4.98; p < 0.001) (Fig 1a). Next, to elucidate what cut-off value is appropriate for the identification of patients with good or poor prognosis, we changed the cut-off value by one point from 14 to 7 points and compared OS between two groups for each cut-off value. The HR for death was the highest value when the cut-off value was set as 14 points (HR, 2.67) (Fig 1a and S1 Fig). In addition, patients with a low G8 score significantly survived less than patients with a high G8 score, regardless of the cut-off value (Fig 1a and S1 Fig). Therefore, we hypothesized that among patients with a G8 score ≤14, patients with a lower score may have poorer prognosis. Then, to elucidate whether subclassification into three groups is more effective for detecting patients with good or poor prognosis than subclassification into two groups, we classified all patients into three groups, the low score group (n = 104), the intermediate score group (n = 115), and the high score group (n = 45), according to their G8 score. Median OS was 7.7 months (95% CI, 5.5–10.7) in the low score group, 15.6 months (95% CI, 10.4–18.1) in the intermediate score group, and 25.6 months (95% CI, 16.4 to not reached) in the high score group. In comparison between any two groups among these three groups, patients with a lower G8 score survived less than patients with a higher G8 score (Fig 1b). The HRs for death were 3.48 (95% CI, 1.97–6.63), 1.83 (95% CI, 1.28–2.65), and 2.09 (95% CI, 1.17–4.02) in comparison between the low and high score groups, the low and intermediate score groups, and the intermediate and high score groups, respectively. Even when all patients enrolled in this study were classified into four groups according to their G8 score, patients with a lower G8 score survived significantly less than patients with a higher G8 score in comparison between any two groups (S2 Fig).

Among the patients enrolled in this study, 187 patients (70.8%) had gastrointestinal carcinomas and 77 patients (29.2%) had the other cancers (malignant tumors other than gastrointestinal carcinoma). We tried to elucidate whether the prediction by the G8 score for prognosis of elderly cancer patients can mainly be applied to patients with gastrointestinal carcinoma or even to patients with other cancers. When classified into three groups according to their G8 score, similar results suggesting that patients with a lower G8 score survived less than patients with a higher G8 score, were observed both in gastrointestinal carcinoma and in the other cancers. Among patients with gastrointestinal carcinoma, median OS was 7.7 months (95% CI, 5.5–10.7) in the low score group (n = 84), 15.6 months (95% CI, 10.9–17.1) in the intermediate score group (n = 76), and 25.6 months (95% CI, 13.3 to not reached) in the high score group (n = 27). Among patients with the other cancers, median OS was 7.0 months (95% CI, 1.6 to not reached) in the low score group (n = 20), 14.2 months (95% CI, 10.0 to not reached) in the intermediate score group (n = 39), and not reached (95% CI, 13.3 to not reached) in the high score group (n = 28) (S3 Fig). These results suggested that the subgrouping by the G8 score may be useful for predicting prognosis of elderly cancer patients with gastrointestinal carcinoma as well as the other cancers.

In our univariate analysis, TNM stage of solid tumor (II/III vs. IV: HR 3.29; 95% CI, 1.65–7.80; p < 0.005), ECOG-PS (0/1 vs. 2/3/4: HR 2.53; 95% CI, 1.64–3.77; p < 0.0001), and the G8 score (the intermediate score vs. the high score: HR 2.09; 95% CI, 1.17–4.02; p < 0.05 and the low score vs. the high score: HR 3.48; 95% CI, 1.97–6.63; p < 0.0001) were significantly associated with OS. In the multivariate analysis, TNM stage (HR 3.59; 95% CI, 1.80–8.52; p < 0.0001) and the G8 score (the intermediate score vs. the high score: HR 1.81; 95% CI, 1.00–3.52; p < 0.05 and the low score vs. the high score: HR 3.34; 95% CI, 1.85–6.47; p < 0.0001) remained independent prognostic factors for OS. However, ECOG-PS had a borderline significant association (HR 1.58; 95% CI, 0.98–2.49; p = 0.06) (Table 4).

**Table 4:** Univariate and multivariate analyses for overall survival.

Factor	No. of patients	Univariate		Multivariate	
		HR (95% CI)	p value	HR (95% CI)	p value
Age					
<80	207	1		1	
≥80	57	1.44 (0.94–2.14)	0.08	1.34 (0.85–2.06)	0.20
Sex					
Male	174	1			
Female	90	1.12 (0.77–1.59)	0.55		
CCI					
0	186	1			
≥1	78	1.33 (0.92–1.90)	0.12		
TNM stage of solid tumor					
II/III	32	1		1	
IV	226	3.29 (1.65–7.80)	< 0.005	3.59 (1.80–8.52)	< 0.0001
ECOG-PS					
0/1	215	1		1	
2/3/4	48	2.53 (1.64–3.77)	< 0.0001	1.58 (0.98–2.49)	0.06
G8 ^a^					
High score	45	1		1	
Intermediate score	115	2.09 (1.17–4.02)	< 0.05	1.81 (1.00–3.52)	< 0.05
Low score	104	3.48 (1.97–6.63)	< 0.0001	3.34 (1.85–6.47)	< 0.0001

Abbreviations: CI, confidential interval; CCI, Charlson comorbidity index; ECOG-PS, Eastern Cooperative Oncology Group performance status.

^a:^ High score, intermediate score, and low score group had a G8 score of 14.5–17, 11–14, and 0–10.5, respectively.

### Prognostic value of G8 for OS in each group classified by ECOG-PS

**Fig 2:**
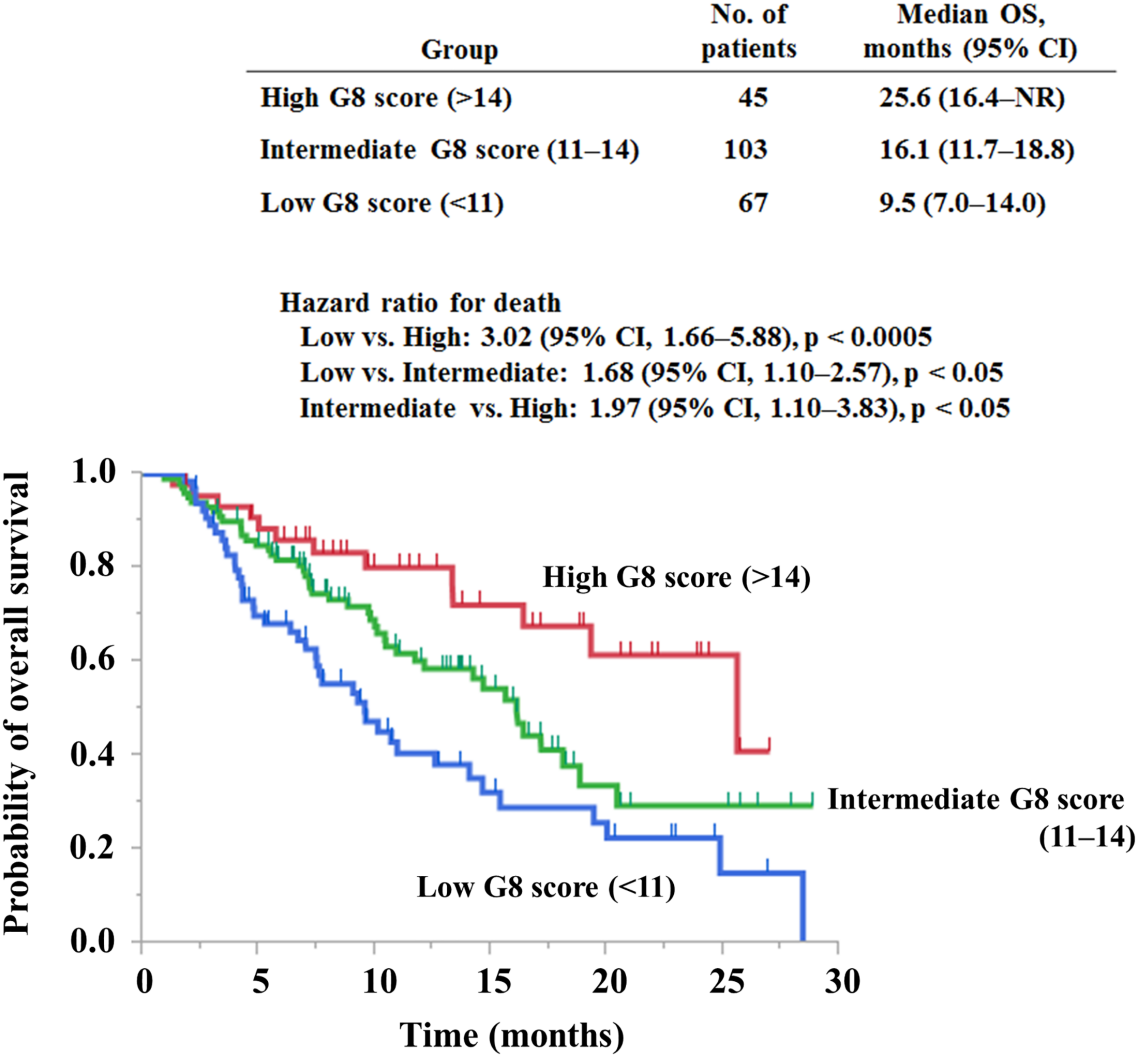
Overall survival according to the G8 score in elderly cancer patients categorized as an ECOG-PS of 0 or 1. Kaplan–Meier analyses for overall survival in patients with high G8 scores (>14), intermediate G8 scores (11–14), or low G8 scores (<11). NR, not reached. ECOG-PS, Eastern Cooperative Oncology Group performance status.

We next evaluated whether our means of G8 score classification into the three groups yielded prognostic value in each group categorized by ECOG-PS. Among 215 patients with an ECOG-PS of 0 or 1, median OS was 9.5 months (95% CI, 7.0–14.0) in the low score group (n = 67), 16.1 months (95% CI, 11.7–18.8) in the intermediate score group (n = 103), and 25.6 months (95% CI, 16.4 to not reached) in the high score group (n = 45) (Fig 2). Patients with intermediate and low G8 scores [(HR 1.97; 95% CI, 1.10–3.83; p < 0.05) and (HR 3.02; 95% CI, 1.66–5.88; p < 0.0005), respectively] survived significantly less than patients with high G8 scores. In addition, comparison between the intermediate score group and the low score group was also significant (HR 1.68; 95% CI, 1.10–2.57; p < 0.05). Patients with a lower G8 score survived less than patients with a higher G8 score in each of the two groups, specifically, in one with an ECOG-PS of 0 or 1 (S4a and S4b Fig).

Among 48 patients with an ECOG-PS of ≥2, no patient was classified into the high G8 score group. Patients with intermediate G8 scores seemed to survive longer compared with patients with low G8 scores, although the difference was not significant (p = 0.45); median OS was 3.9 months (95% CI, 2.2–15.4) in the low score group (n = 37) and 10.0 months (95% CI, 1.4 to not reached) in the intermediate score group (n = 11) (S5 Fig).

### Subgroup analyses for OS in patients with stage IV gastrointestinal carcinoma

Among our cohort of patients enrolled in this study, 162 patients (61.4%) had stage IV gastrointestinal carcinoma, including esophageal, gastric, duodenal, colorectal, pancreatic, and bile tract carcinoma. We then next attempted to analyze the association between G8 score and OS in elderly patients with stage IV gastrointestinal carcinoma. Among 162 patients, 23 patients (14.2%) with a normal G8 score showed median OS of 25.6 months (95% CI, 13.3 to not reached), and 139 patients (85.8%) with an abnormal G8 score showed that of 9.2 months (95% CI, 7.2–12.1). The HR for death was 2.66 (95%CI, 1.36–6.01; p < 0.01) (S6a Fig). The results of analyses for OS in the three groups categorized according to the G8 score are shown in S6b Fig. Median OS was 7.4 months (95% CI, 4.3–9.2) in the low score group (n = 71), 14.6 months (95% CI, 8.8–16.4) in the intermediate score group (n = 68), and 25.6 months (95% CI, 13.3 to not reached) in the high score group (n = 23). In comparison between any two groups among these three groups, patients with a lower G8 score significantly survived less than patients with a higher G8 score (S6b Fig).

## Discussion

This study is the first report that the novel G8 classification into three groups has led to more efficient identification of patients with poor prognosis than the conventional classification into two groups. Via this method, we found that G8 enhances the prognostic value of ECOG-PS in elderly patients with advanced cancer.

Bellera et al. defined a G8 score of 14 as the optimal cut-off value for identifying elderly cancer patients requiring a full GA [5]. In our study, 83% of patients had a G8 score of ≤14, namely abnormal. The proportion of patients with an abnormal G8 score was slightly higher than or equal to previous studies showing that 61–86% of elderly cancer patients had an abnormal G8 score [5–7, 12, 13, 20]. Approximately 90% of all patients in our study had TNM stage IV cancer, whereas 52% in Kenis’ study [6], 17% and 48% had metastatic lesions in Soubeyran’s study [7] and Liuu’s study [20], respectively. In addition, more than 70% of all patients in our study had gastrointestinal carcinoma, whereas 21% had colorectal carcinoma in Kenis’ study [6] and less than 20% and 38% had gastrointestinal carcinoma in Soubeyran’s study [7] and in Liuu’s study [20], respectively. Patients with more advanced cancer, particularly gastrointestinal carcinoma, as compared with other cancers, tended to have worse general conditions regarding nausea and anorexia, which thereafter led to less food intake, body weight loss, lower body mass index, more prescription drugs, and lower self-perception of health. We speculated that for this reason, most patients (83%) had an abnormal G8 score, with a low score for each of the five items assessed in this study.

In several previous studies that analyzed the association between the G8 score and OS, the cut-off value of G8 was set as 14 points [6, 12, 13, 21]. Kenis et al. reported that patients with a G8 score of ≤14 significantly survived less than patients with a score of >14 in 937 elderly patients with various types of cancer [6]. Similarly, it was reported that patients with a G8 score of ≤14 had a significantly higher risk of mortality at one year than patients with a score of >14 as noted in Denewet’s study that included 205 elderly patients with various cancers [12] and in Hamaker’s study that included 108 elderly patients with hematological malignancy [13]. In our study, when all patients were newly classified into three, or even four groups, the lower score group had significantly worse survival compared with higher score group. This new subclassification of the G8 score may lead to more efficient identification of elderly cancer patients with poor prognosis.

In general, patients with more advanced cancer, particularly a terminal stage of cancer, may have lower G8 score as well as poorer ECOG-PS. Therefore, one might suspect that both the lower G8 score and the poorer ECOG-PS are equally associated with worse survival. However, our multivariate analysis, which included age, TNM stage, ECOG-PS, and the G8 score as covariates, has demonstrated that G8 had better prognostic value than ECOG-PS as shown in Table 4. Several studies have reported the association between the full GA and OS of elderly cancer patients [2, 3, 22]. Although cancer type, clinical stage, and treatment varied in each study, it was reported that functional status [23–25], nutritional status [26–28], mental status [27, 29, 30], polypharmacy [30], and comorbidity [29, 31, 32] were independent predictors for OS. G8 is simplified by extracting seven items from the MNA, which is one of the GA tools used in evaluating for nutritional status. Different from ECOG-PS, G8 includes items to evaluate not only for functional status but also for nutrition, neuropsychological status, and polypharmacy. Consequently, G8 has good prognostic value for OS, possibly because G8 confers the advantage of evaluating general conditions of patients via multiple aspects.

Eighty-one percent of all patients had an ECOG-PS of 0 or 1 in our study. Although these patients generally tolerate standard therapy better and survive longer than patients with an ECOG-PS of ≥2, our results demonstrated that patients with an ECOG-PS of 0 or 1, but with a low G8 score, survived less. Thus, evaluating G8 in elderly cancer patients with good functional status as categorized with an ECOG-PS of 0 or 1 could also contribute to enhanced detection of patients with poor prognosis. Our results also support that the G8 screening tool can provide additional information that cannot simply be obtained by the assessment for functional status alone, such as that using ECOG-PS.

There is a growing need for an appropriate diagnostic tool for physicians to select, among elderly cancer patients, who should receive a standard therapy that are basically provided to young patients, who should receive an attenuated therapy, or who should not receive any therapy. We propose one possible strategy that elderly cancer patients with a low G8 score, regardless of ECOG-PS, considered to have shorter survival and to be intolerable to an intensive therapy, should receive an attenuated therapy or no therapy. Whether our new G8 subclassification can be useful for deciding treatment options for elderly cancer patients should be validated in future prospective clinical trials. When validated, the G8 screening tool can be a helpful diagnostic method for physicians, as well as elderly cancer patients and their family, to choose appropriate treatments.

There were some study limitations. Firstly, this study was a retrospective and performed at a single institution. Secondly, this study included various cancers, such as head and neck carcinoma, carcinoma of unknown primary, sarcoma, and hematological malignancy, but a majority was gastrointestinal carcinoma. It should be noted that there may be a concern as to whether our main conclusion can be applied to other cancers. Nevertheless, our results support the usefulness of G8 for predicting prognosis of patients with the other cancers as well as gastrointestinal carcinoma as shown in S3 Fig. In addition, the HR for death when all patients were classified into two groups with a cut-off value of 14 was 2.67 in our study, which was approximately equivalent to that of 2.63 in Kenis’ study that included breast (40%), colorectal (21%), prostate (9%), lung (8%), and ovarian carcinoma (6%), and hematological malignancy (16%) [6]. Thus, our corroborative findings may support the notion that our work herein is applicable to other types of cancer.

In conclusion, we have shown that the new G8 classification scheme as divided into three subgroups and the combination of ECOG-PS and G8 are useful prognostic factors for elderly patients with advanced cancer. The G8 screening tool may help physicians make informed treatment decisions. A large-scale prospective study is warranted to further detail the value of G8 as a prognostic and/or predictive marker for treatments concerning elderly cancer patients.

## Supporting information

S1 Fig Overall survival according to the G8 score with the various cut-off values in elderly cancer patients. (PDF)

S2 Fig Overall survival in elderly cancer patients in classification into four groups according to the G8 score. (PDF)

S3 Fig Overall survival according to the G8 score in elderly patients with gastrointestinal carcinoma and the other cancers. (PDF)

S4 Fig Overall survival according to the G8 score in each group, an ECOG-PS of 0 or 1. (PDF)

S5 Fig Overall survival according to the G8 score in elderly cancer patients categorized as an ECOG-PS of ≥2. (PDF)

S6 Fig Overall survival according to the G8 score in elderly patients with stage IV gastrointestinal carcinoma. (PDF)

S1 Table The G8 screening tool. (PDF)

S2 Table Clinicopathological data of 264 patients enrolled in this study. (XLSX)

S3 Table Score in each item. (PDF)
