# Supplementary material for: The G8 screening tool enhances prognostic value to ECOG performance status in elderly cancer patients: A retrospective, single institutional study
Source: PLoS One. 2017 Jun 22;12(6):e0179694. doi: 10.1371/journal.pone.0179694 (PMC5480957; doi:10.1371/journal.pone.0179694)
Supplement: S1 Fig — (PDF) [file pone.0179694.s001.pdf]

Supporting Figure 1

a

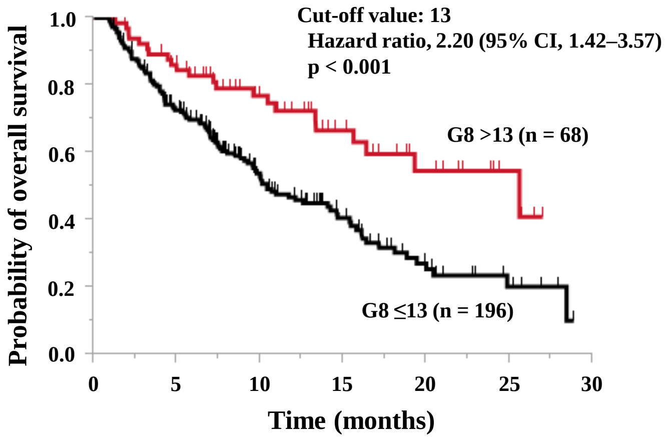

b

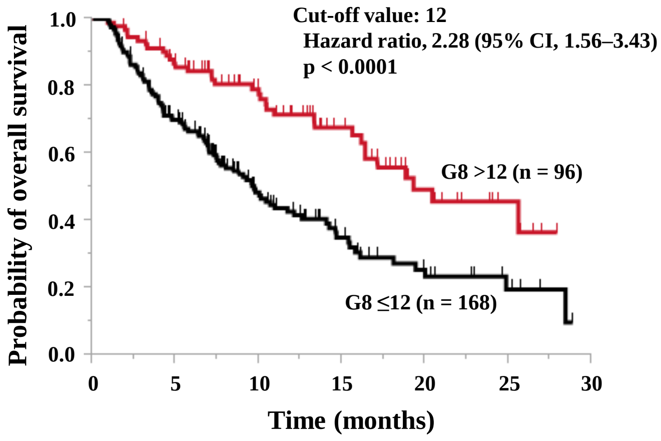

c

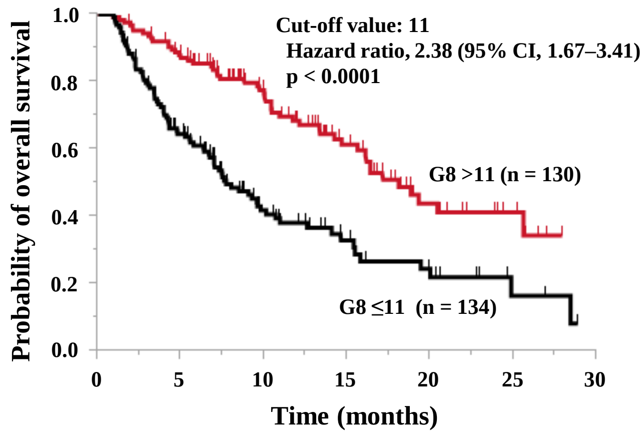

d

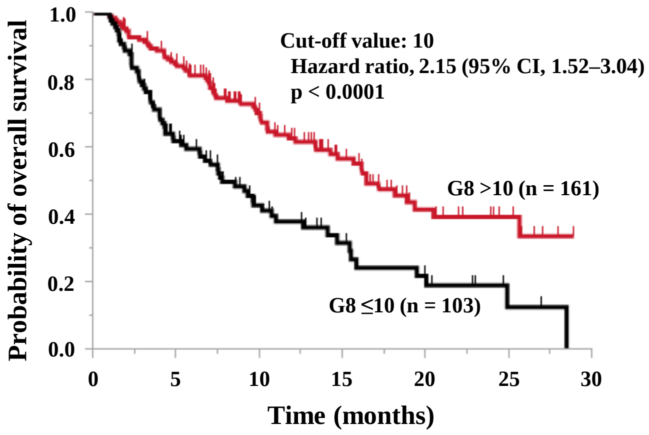

e

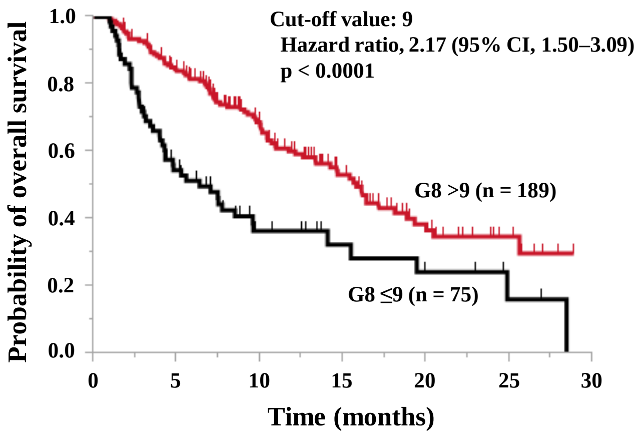

f

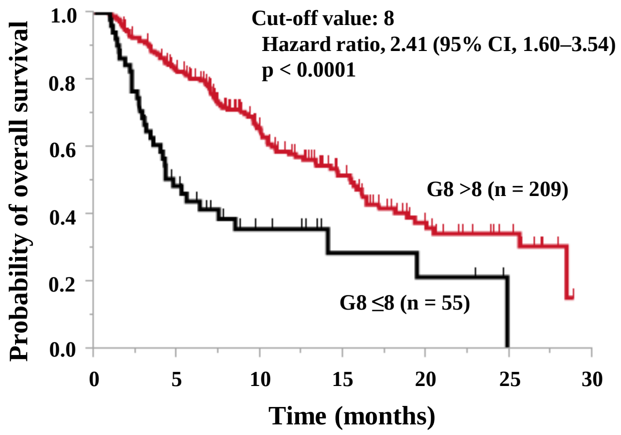

g

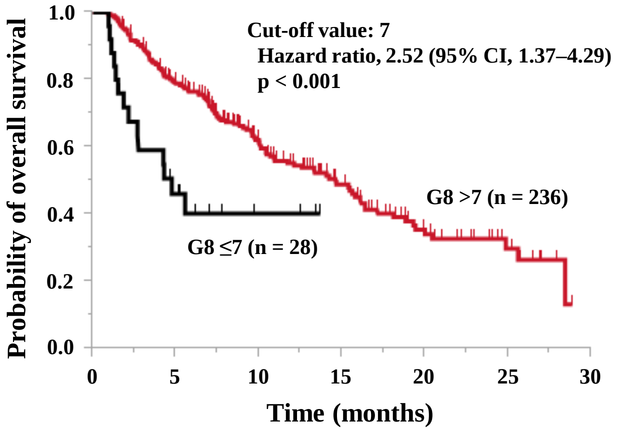

Supporting Figure 1:
Overall survival according to the G8 score with the various cut-off values in elderly cancer patients.
Kaplan–Meier analyses for overall survival are shown. The cut-off value of G8 is 13 points in (a), 12 in (b), 11 in (c), 10 in (d), 9 in (e), 8 in (f), and 7 in (g).
