# Supplementary material for: The G8 screening tool enhances prognostic value to ECOG performance status in elderly cancer patients: A retrospective, single institutional study
Source: PLoS One. 2017 Jun 22;12(6):e0179694. doi: 10.1371/journal.pone.0179694 (PMC5480957; doi:10.1371/journal.pone.0179694)
Supplement: S2 Fig — (PDF) [file pone.0179694.s002.pdf]

Supporting Figure 2

Group	No. of patients	Median OS, months (95% CI)
G8 score: 14.5–17.0	45	25.6 (16.4–NR)
G8 score: 11.5–14	85	16.1 (11.7–18.8)
G8 score: 8.5–11	79	9.6 (7.3–14.6)
G8 score: 0–8	55	4.7 (3.4–8.5)

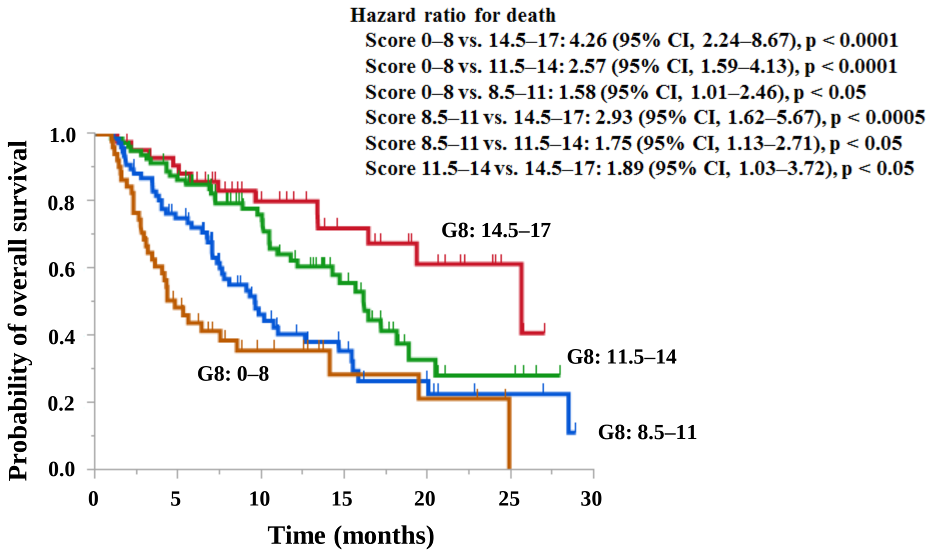

Supporting Figure 2:
Overall survival in elderly cancer patients in classification into four groups according to the G8 score.
Kaplan–Meier analyses for overall survival are shown. NR, not reached.
