# Supplementary material for: The G8 screening tool enhances prognostic value to ECOG performance status in elderly cancer patients: A retrospective, single institutional study
Source: PLoS One. 2017 Jun 22;12(6):e0179694. doi: 10.1371/journal.pone.0179694 (PMC5480957; doi:10.1371/journal.pone.0179694)
Supplement: S3 Fig — (PDF) [file pone.0179694.s003.pdf]

Supporting Figure 3

a

Group	No. of patients	Median OS, months (95% CI)
High G8 score (>14)	27	25.6 (13.3–NR)
Intermediate G8 score (11–14)	76	15.6 (10.9–17.1)
Low G8 score (<11)	84	7.7 (5.5–10.7)

Hazard ratio for death
Low vs. High: 3.19 (95% CI, 1.63–7.00), p < 0.005
Low vs. Intermediate: 1.74 (95% CI, 1.14–2.69), p < 0.05
Intermediate vs. High: 1.99 (95% CI, 0.98–4.45), p = 0.07

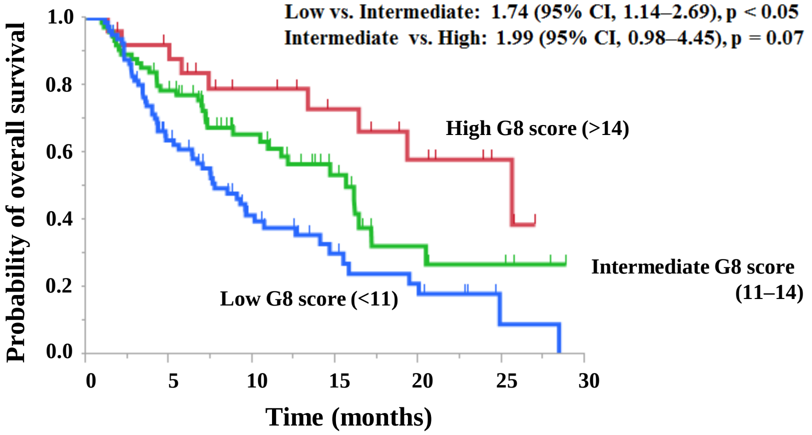

b

Group	No. of patients	Median OS, months (95% CI)
High G8 score (>14)	18	NR (13.3–NR)
Intermediate G8 score (11–14)	39	14.2 (10.0–NR)
Low G8 score (<11)	20	7.0 (1.6–NR)

Hazard ratio for death
Low vs. High: 4.07 (95% CI, 1.38–14.78), p < 0.05
Low vs. Intermediate: 2.01 (95% CI, 0.91–4.26), p = 0.07
Intermediate vs. High: 2.28 (95% CI, 0.84–7.94), p = 0.13

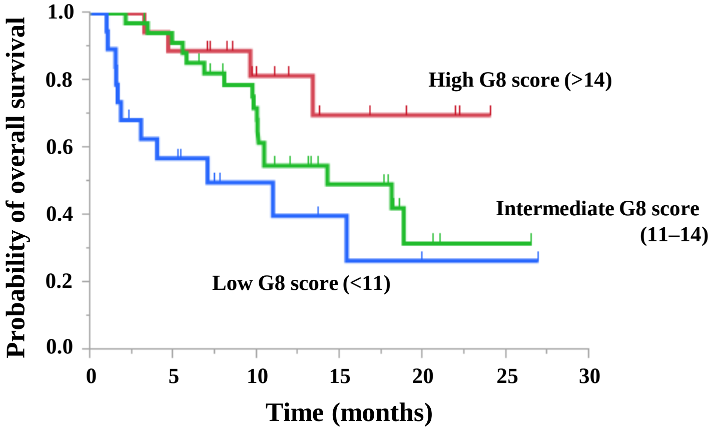

Supporting Figure 3:
Overall survival according to the G8 score in elderly patients with gastrointestinal carcinoma and the other cancers.
Kaplan–Meier analyses for overall survival in patients with gastrointestinal carcinoma and the other cancers in (a) and (b), respectively. Patients were classified into three groups, the high score, the intermediate score, or the low score, according to their G8 score. NR, not reached.
