# Supplementary material for: The G8 screening tool enhances prognostic value to ECOG performance status in elderly cancer patients: A retrospective, single institutional study
Source: PLoS One. 2017 Jun 22;12(6):e0179694. doi: 10.1371/journal.pone.0179694 (PMC5480957; doi:10.1371/journal.pone.0179694)
Supplement: S4 Fig — (PDF) [file pone.0179694.s004.pdf]

Supporting Figure 4

a

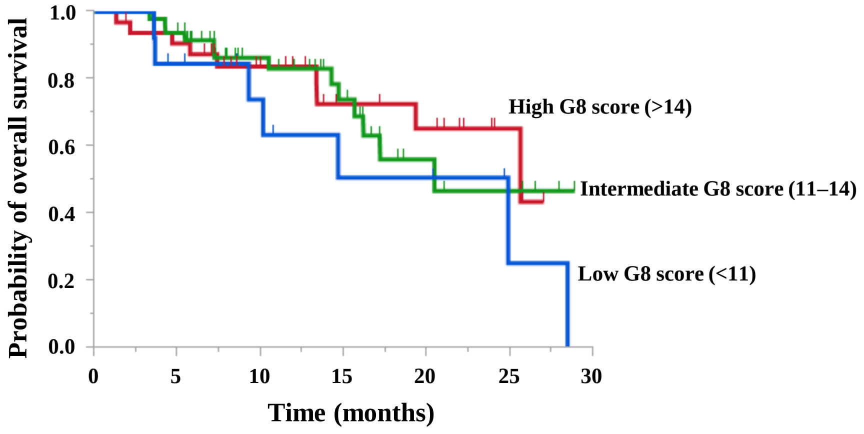

b

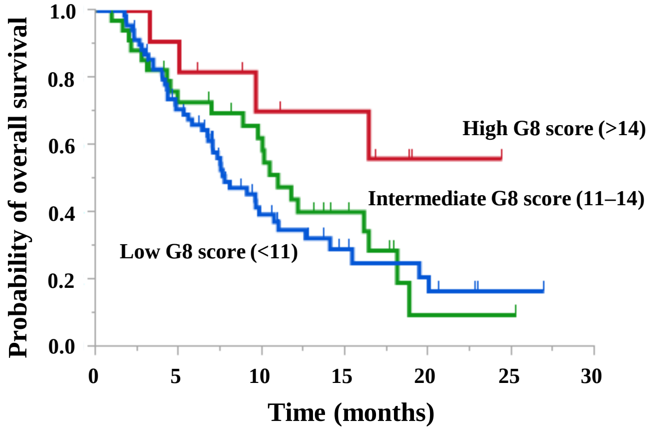

Supporting Figure 4:
Overall survival according to the G8 score in each group, an ECOG-PS of 0 or 1.
Kaplan–Meier analyses for overall survival in patients with an ECOG-PS of 0 and with an ECOG-PS of 1 are shown in (a) or (b), respectively. Patients were classified into three groups, the high score, the intermediate score, or the low score, according to their G8 score. ECOG-PS, Eastern Cooperative Oncology Group performance status.
