# Supplementary material for: The G8 screening tool enhances prognostic value to ECOG performance status in elderly cancer patients: A retrospective, single institutional study
Source: PLoS One. 2017 Jun 22;12(6):e0179694. doi: 10.1371/journal.pone.0179694 (PMC5480957; doi:10.1371/journal.pone.0179694)
Supplement: S5 Fig — (PDF) [file pone.0179694.s005.pdf]

Supporting Figure 5

Group	No. of patients	Median OS, months (95% CI)
Intermediate G8 score (11–14)	11	10.0 (1.4–NR)
Low G8 score (<11)	37	3.9 (2.2–15.4)

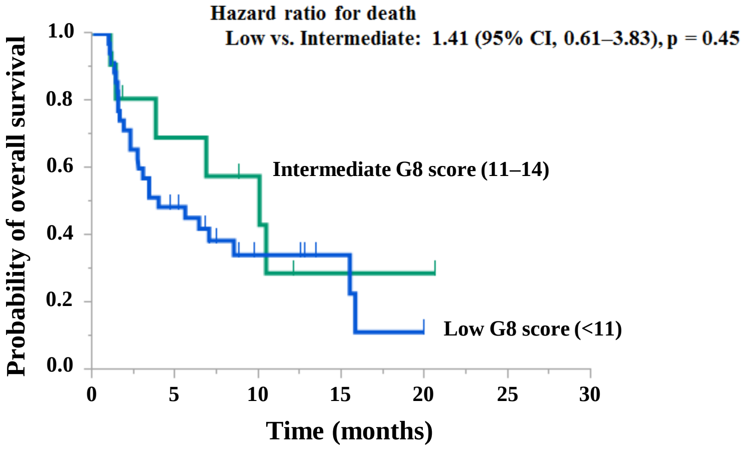

Supporting Figure 5:
Overall survival according to the G8 score in elderly cancer patients categorized as an ECOG-PS of ≥2.
Kaplan–Meier analyses for overall survival are shown. Although patients were classified into three groups, the high score, the intermediate score, or the low score, according to their G8 score, no patient with an ECOG-PS of ≥2 was classified into the high G8 score group. ECOG-PS, Eastern Cooperative Oncology Group performance status.
