# Supplementary material for: The G8 screening tool enhances prognostic value to ECOG performance status in elderly cancer patients: A retrospective, single institutional study
Source: PLoS One. 2017 Jun 22;12(6):e0179694. doi: 10.1371/journal.pone.0179694 (PMC5480957; doi:10.1371/journal.pone.0179694)
Supplement: S6 Fig — (PDF) [file pone.0179694.s006.pdf]

Supporting Figure 6

a

Group	No. of patients	Median OS, months (95% CI)
Normal G8 score (>14)	23	25.6 (13.3–NR)
Abnormal G8 score (≤14)	139	9.2 (7.2–12.1)

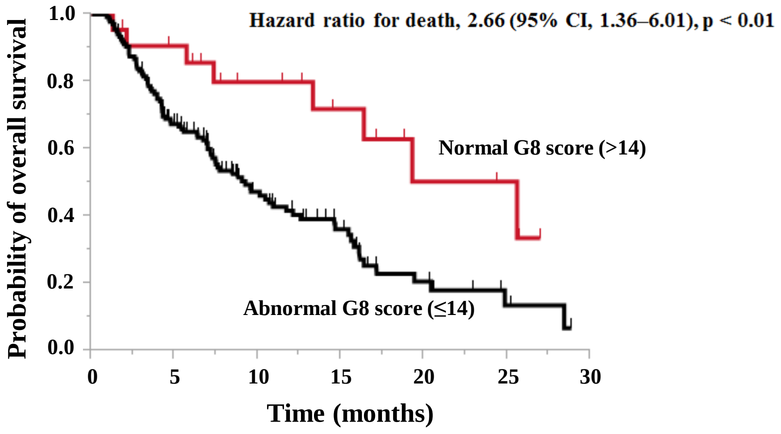

b

Group	No. of patients	Median OS, months (95% CI)
High G8 score (>14)	23	25.6 (13.3–NR)
Intermediate G8 score (11–14)	68	14.6 (8.8–16.4)
Low G8 score (<11)	71	7.4 (4.3–9.2)

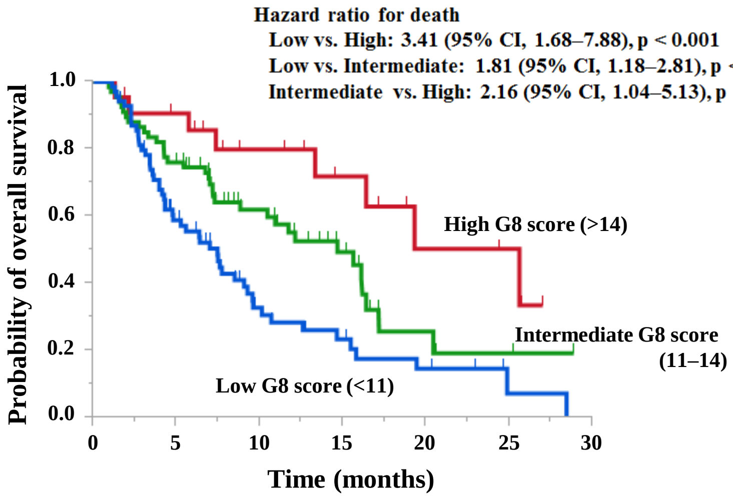

Supporting Figure 6:
Overall survival according to the G8 score in elderly patients with stage IV gastrointestinal carcinoma.
(a) Kaplan–Meier analyses for overall survival in patients with a normal G8 score (>14) or an abnormal G8 score (≤14).
(b) Kaplan–Meier analyses for overall survival in patients with high G8 scores (>14), intermediate G8 scores (11–14), or low G8 scores (<11). NR, not reached.
