# Supplementary material for: The G8 screening tool enhances prognostic value to ECOG performance status in elderly cancer patients: A retrospective, single institutional study
Source: PLoS One. 2017 Jun 22;12(6):e0179694. doi: 10.1371/journal.pone.0179694 (PMC5480957; doi:10.1371/journal.pone.0179694)
Supplement: S1 Table — (PDF) [file pone.0179694.s007.pdf]

Supporting Table 1. The G8 screening tool.

Item	Score
1: Has food intake declined over the past 3 months due to loss of appetite, digestive problems, chewing or swallowing difficulties?	0 = severe decrease in food intake
	1 = moderate decrease in food intake
	2 = no decrease in food intake
2: Weight loss during the last 3 months	0 = weight loss greater than 3 kg
	1 = does not know
	2 = weight loss between 1 and 3 kg
	3 = no weight loss
3: Mobility	0 = bed or chair bound
	1 = able to get out of bed/chair but does not go out
	2 = goes out
4: Neuropsychological problems	0 = severe dementia or depression
	1 = mild dementia
	2 = no psychological problems
5: BMI = weight in kg/(height in m)^2	0 = BMI less than 19
	1 = BMI 19 to less than 21
	2 = BMI 21 to less than 23
	3 = BMI 23 or greater
6: Takes more than 3 prescription drugs per day	0 = yes
	1 = no
7: In comparison with other people of the same age, how does the patient consider his/her health status?	0 = not as good
	0.5 = does not know
	1 = as good
	2 = better
8: Age	0 = >85 years
	1 = 80–85 years
	2 = <80 years

Abbreviation: BMI, body mass index.
