# Supplementary material for: The G8 screening tool enhances prognostic value to ECOG performance status in elderly cancer patients: A retrospective, single institutional study
Source: PLoS One. 2017 Jun 22;12(6):e0179694. doi: 10.1371/journal.pone.0179694 (PMC5480957; doi:10.1371/journal.pone.0179694)
Supplement: S3 Table — (PDF) [file pone.0179694.s009.pdf]

**Supporting Table 3.** Score in each item.

| Item, score | No. of patients (n = 264) | % |
|---|---|---|
| 1: Food intake | | |
| 0 | 55 | 20.8 |
| 1 | 78 | 29.5 |
| 2 | 131 | 49.6 |
| 2: Weight loss | | |
| 0 | 111 | 42.0 |
| 1 | 5 | 1.9 |
| 2 | 43 | 16.3 |
| 3 | 105 | 39.8 |
| 3: Mobility | | |
| 0 | 10 | 3.8 |
| 1 | 45 | 17.0 |
| 2 | 209 | 79.2 |
| 4: Neuropsychological problem | | |
| 0 | 7 | 2.7 |
| 1 | 10 | 3.8 |
| 2 | 247 | 93.6 |
| 5: Body mass index | | |
| 0 | 54 | 20.5 |
| 1 | 71 | 26.9 |
| 2 | 58 | 22.0 |
| 3 | 81 | 30.7 |
| 6: Prescription drug | | |
| 0 | 173 | 65.5 |
| 1 | 91 | 34.5 |
| 7: Self-perception of health | | |
| 0 | 82 | 31.1 |
| 0.5 | 19 | 7.2 |
| 1 | 72 | 27.3 |
| 2 | 91 | 34.5 |
| 8: Age | | |
| 0 | 8 | 3.0 |
| 1 | 49 | 18.6 |
| 2 | 207 | 78.4 |

Higher score in each item indicates better condition.
